# Overcome of Carbon Catabolite Repression of Bioinsecticides Production by Sporeless *Bacillus thuringiensis* through Adequate Fermentation Technology

**DOI:** 10.1155/2014/698587

**Published:** 2014-09-17

**Authors:** Saoussen Ben Khedher, Samir Jaoua, Nabil Zouari

**Affiliations:** ^1^Team of Biopesticides (LPIP), Centre of Biotechnology of Sfax, Sfax University, 3018 Sfax, Tunisia; ^2^Department of Biological & Environmental Sciences, College of Arts and Sciences, Qatar University, P.O. Box 2713, Doha, Qatar

## Abstract

The overcoming of catabolite repression, in bioinsecticides production by sporeless *Bacillus thuringiensis* strain S22 was investigated into fully controlled 3 L fermenter, using glucose based medium. When applying adequate oxygen profile throughout the fermentation period (75% oxygen saturation), it was possible to partially overcome the catabolite repression, normally occurring at high initial glucose concentrations (30 and 40 g/L glucose). Moreover, toxin production yield by sporeless strain S22 was markedly improved by the adoption of the fed-batch intermittent cultures technology. With 22.5 g/L glucose used into culture medium, toxin production was improved by about 36% when applying fed-batch culture compared to one batch. Consequently, the proposed fed-batch strategy was efficient for the overcome of the carbon catabolite repression. So, it was possible to overproduce insecticidal crystal proteins into highly concentrated medium.

## 1. Introduction

During the last decades, the use of* Bacillus thuringiensis* bioinsecticides became more preferable for pest managements than conventional chemical ones, due to their lower pollution and their biodegradability. The spore-forming bacterium* B. thuringiensis *produces crystalline inclusions which are highly effective and specific to Lepidoptera, Coleopteran, and Diptera insects' larvae [1]. Interestingly, the use of sporeless* B. thuringiensis* strain, which is more environmentally friendly, is an attractive alternative in pest management [2]. Several mutants, deficient in sporulation, were shown to be hyperproducers of delta-endotoxins [2]. So, the improvement of* B. thuringiensis *bioinsecticides production by such strains became a priority in the development at a large scale up. For* B. thuringiensis* isolates, this was achieved through application of mutagenesis [2, 3], adaptation to abiotic stress conditions [4, 5], using appropriate media [6–8], overcoming of metabolic limitations [9], and application of an adequate fermentation technology [10]. Since the primary interest of fermentation research is the cost-effective production of bioproducts, it is important to develop a cultivation method that allows bioinsecticides production at high concentration with high production yield. Nevertheless, the catabolite repression is one of the major metabolic limitations of delta-endotoxin production [9]. In fact, the adequate control of dissolved oxygen and the good fed-batch strategy allowed at least partial overcome of such limitation [11]. Several fed-batch strategies for* B. thuringiensis* were reported. These strategies involved glucose feeding [12, 13], hybrid neural network control [14], or motile intensity controls [15]. Glucose played roles as energy source, carbon source in delta-endotoxin synthesis and the glucose feeding is regarded as the most important factor to increase the final yield. In a previously reported work [2], overproducing sporeless (asporogenic and oligosporogenic) mutants were shown to overproduce delta-endotoxins compared to sporulating wild strain. In this study, the overcome of catabolite repression was investigated by the optimization of the adequate control of dissolved oxygen in the culture medium and by the development of a new fed-batch strategy based on the analysis of the glucose and product variation in the batch culture and in the fed-batch intermittent culture (FBIC) of* B. thuringiensis*. An asporogenic* B. thuringiensis* strain S22 was used in this work.

## 2. Materials and Methods

### 2.1. Strain

The sporeless* B. thuringiensis* subsp* kurstaki *strain S22 [2] was used as a representative strain for the study.

### 2.2. Inocula Preparation

The inocula were prepared as reported by Ghribi et al. [3]: one isolated colony was dispensed in 3 mL of LB medium and incubated overnight at 30°C. Aliquots (0.5 mL) were used to inoculate 1000 mL flask containing 100 mL LB medium. After 6 h of incubation at 30°C in a rotary shaker set at 200 rpm, the absorbance at 600 nm was determined. The culture broth was used to inoculate the studied media to start with an initial O.D.600 of 0.15.

### 2.3. Culture Medium

The optimized culture medium previously described by Ben Khedher et al. [8] was used with the following composition (g/L): glucose, 22.5; glycerol, 4.8; yeast extract, 5.8; ammonium sulphate, 5.4; KH_2_PO_4_, 1; K_2_HPO_4_, 1; MgSO_4_, 0.3; MnSO_4_, 0.008, and FeSO_4_, 0.01. Medium pH was adjusted to 7.0 before sterilization at 121°C for 20 min.

### 2.4. Bioinsecticides Production into 3 L Fermenter

Production experiments were carried out at 30°C into 3 L Labfors (Infors, Switzerland) full automatically controlled fermenter containing 1.7 L culture medium with continuous regulation of pH using 2 N HCl and 2 N NaOH. During the fermentation process, a different oxygenation level was maintained. The studied aeration profiles (30%, 40%, 50%, 60%, 70%, and 75% dissolved oxygen saturation) were performed by combination of aeration rates (ranged from 0.5 to 5.0 vvm) and agitation speeds (ranged from 200 to 1500 rpm). It was continuously monitored by an oxygen sensor (InPro 6000 Oxygen sensor, Mettler Toledo, Switzerland). Foaming was controlled by the use of an antifoam (Struktol SB2020, Schills seilacher, Hamburg Germany), through the fermentation.

### 2.5. Full Factorial Design

Prior experiments revealed that aeration profile, glucose, and yeast extract concentrations may have a high impact on delta-endotoxins production. A 2^3^ factorial design was done to evaluate the significance of these factors. The variables and their levels were reported in Table 1.

### 2.6. Fed-Batch Regime

In this study, fed-batch strategy was conducted based on residual glucose consumption in the medium as control parameter and using manual intermittent feed mode to feed the fermenter. Fed-batch cultures were started from 2 to 10 h of fermentation and the feed rate was determined based on glucose consumption measurement during the exponential phase growth. During the first ten hours of culture, the consumption of glucose in the fermenter was measured by the determination of residual glucose in the sample medium withdrawn each 2 h and the consumed glucose was fed with a concentrated glucose solution (200 g/L) to maintain a steady glucose concentration in the medium.

### 2.7. Delta-Endotoxin Determination

Delta-endotoxin concentration was determined in the solubilized crystal preparation from each culture medium as described by Zouari et al. [9]. In summary, 1 mL of culture medium was centrifuged for 10 min at 10,000 ×g and the pellet was washed twice with 1 M NaCl and twice with distilled water. The pellet was suspended in 1 mL of 50 mmol/L NaOH (pH 12.5) in order to solubilize delta-endotoxin crystals. After 2 h of incubation at 30°C, a total protein in the supernatant was measured by using Bio-Rad reagent (Bio-Rad Protein assay, cat. 500–0006) according to Bradford method [16].

### 2.8. Biomass Determination

The number of cells was estimated by counting CFU at the end of the experiments. Cells from postlogarithmic phase of growth were plated onto LB agar and incubated at 30°C for 24 h.

### 2.9. Residual Glucose Concentration Determination

The glucose was monitored, in the supernatant of S22 culture media, by using Glucose Oxydase (Biomaghreb, Tunisia, GOD, PAL) according to the method of Lloyd and Whelan [17].

### 2.10. Bioassays

Bioassays were carried out using third instar larvae of* Ephestia kuehniella *(*E. kuehniella*) kindly provided by the Institute of the Olive Tree, Sfax, Tunisia. As described by Tounsi et al. [18], ten larvae were transferred to sterile bottles containing flour mixed with delta-endotoxins at desired concentrations. Each test was repeated in triplicate. Mortality was recorded after 4 days at 28°C. Fifty percent lethal concentration (LC_50_) for each strain was calculated from pooled raw data by probit analysis using programs written in the* R*. language [19].

### 2.11. Statistical Analysis

All the results related to determination of delta-endotoxin production, CFU counts, and residual glucose amounts were the average of three replicates for each cultural condition. They were statistically analyzed by SAS software (version 6) using Duncan test performed after analysis of variance (ANOVA).

## 3. Results and Discussion

### 3.1. Involvement of Dissolved Oxygen in Bioinsecticides Production in Glucose Based Medium

In order to study the effect of oxygen on growth and particularly on delta-endotoxin synthesis by the sporeless strain S22, experiments were performed and the corresponding fermentation profiles and the detailed data were collected and shown in Table 2. The highest delta-endotoxin production was reached at an oxygen profile corresponding to 60% oxygen saturation during the first 6 h of incubation followed by reduction down to 40% oxygen saturation, up to the end of fermentation. When dissolved oxygen saturation was increased from 60% to 70% during the first 6 h of growth, biomass production was statistically similar with decrease in toxin production and yield. With a lower aeration (40% oxygen saturation) throughout the fermentation, the toxin synthesis was significantly reduced (Table 2). A high aeration profile (60%–50%) throughout the fermentation conducted to a low delta-endotoxin synthesis (3430 mg) and consequently a decrease in toxin synthesis yield (114.33 mg toxin/10^10^ cells). When dissolved oxygen rate was reduced from 60% to 40%, it did not affect cell growth but it has an impact on delta-endotoxin synthesis which reached an optimal level of 4338 mg and a synthesis yield of 120.5 mg toxin/10^10^ cells (Table 2). This means that the delta-endotoxin production and the toxin synthesis yield by cells were increased when compared to the other aeration profiles.

These results showed that in 3 L fermenter, the dissolved oxygen requirements during each fermentation phase played a key role in delta-endotoxin production. In fact, the control of dissolved oxygen appeared to be necessary during the first 6 h of vegetative growth for regulating the carbon source assimilation rate. The need to decrease oxygen saturation after 6 h of vegetative growth down to 40% could be explained by additional time for* cry *gene expression and crystal formation [6]. Previously, optimizing the adequate concentration of dissolved oxygen has been reported as an important factor in the batch culture of* B. thuringiensis* [20, 21]. Although there were several studies available in relation to the effects of oxygen on the biomass concentration [22], there were few works concerning the effects of oxygen on toxin synthesis [23]. Moreover, investigations were carried out under constant aeration conditions, throughout the fermentation. However, the oxygen demands of culture were not the same throughout the culture and depended on the physiological state of the cell in the culture, as demonstrated in our studies. The variability in delta-endotoxin production noted under the different aeration profiles suggests that delta-endotoxin synthesis by sporeless* B. thuringiensis* S22 depended on a suitable oxygen supply to the cells. To the contrary, cell growth did not increase or decrease significantly when different aeration profiles were tested. So, there was no relationship or correlation between biomass and delta-endotoxin production. However, the oxygen availability to the medium was essential to guarantee optimal* B. thuringiensis* bioinsecticides production. Nevertheless, this factor must be carefully controlled since the excess or the limitation of oxygen can affect the metabolic pathways, involved in delta-endotoxin production.

Previously, several studies were carried out and gave improvements to batch culture conditions for increasing spore and/or delta-endotoxin production [24]. Some reports were interested in the optimization of nutritional and cultural factors for* B. thuringiensis* subsp.* israelensis* HD 500 crystal toxin components Cry4Ba and Cry11Aa, such as Mn level, K_2_HPO_4_ level, C : N ratio, and incubation temperature [25]. The optimized toxins productions were 28.9 mg/L for Cry4Ba and 69.2 mg/L for Cry11Aa. Nevertheless,* B. thuringiensis* subsp.* israelensis* delta-endotoxins production was 415 mg/L through optimization of dissolved oxygen level in the culture medium [26], which was 5 folds lower than S22 delta-endotoxins production (2169 mg/L, Ben Khedher, S., unpublished data). So, considering the high production and stability of bioinsecticides based on sporeless* B. thuringiensis,* S22 strain is a promising strain for practical applications. The bioassay results against* E. kuehniella* larvae showed a significant increase in LC_50_ value, at high (60–50% oxygen saturation) and low (40% oxygen saturation) aeration levels throughout the fermentation (Table 2). Nevertheless, the highest delta-endotoxin production was associated with a significant decrease in LC_50_ value, which implied an increase in insecticidal activity. Taking into account all effects of oxygen, it can be concluded that carrying out high aeration level during the first 6 h of culture (60% oxygen saturation) followed by a moderate oxygenation rate (30 or 40% oxygen saturation) up to the end of fermentation could induce an increase in delta-endotoxin production and an improve in* B. thuringiensis *toxicity.

### 3.2. Overcome of Catabolite Repression on Delta-Endotoxin Synthesis by Optimization of Aeration Level into 3 L Fermenter

Several studies reported that high concentration of glucose inhibits the growth of* B. thuringiensis*, harming the sporulation and toxin synthesis [27]. Besides, experiments carried out into 1000 mL shake flasks confirmed that, at high concentrations, glucose exhibited repressive effect on delta-endotoxin synthesis (data not shown). In order to overcome the catabolite repression which is considered as the major limitation of delta-endotoxin synthesis [9], a 2^3^ factorial design with three factors (aeration profiles, glucose, and yeast extract concentrations) at two levels, 8 experimental runs were performed in triplicate.  Table 3 shows the three independent variables and their values of the factorial design experiments. Delta-endotoxin yield production decreased from 113.41 mg/g glucose (Table 2) to 78.52 mg/g glucose (Table 3) when increasing glucose concentration from 22.5 g/L to 30 g/L, at 60% oxygen saturation during the first 6 h followed by reduction down to 40% oxygen saturation, up to the end of fermentation. Interestingly, with 75% oxygen saturation in the medium throughout the fermentation, an improvement of delta-endotoxin production was noticed and was associated to an improvement in yield production which reached 95.5 mg/g glucose (Table 3). Providing dissolved oxygen at 75% oxygen saturation in the medium throughout the fermentation was recommended only at high carbon source concentration. The analysis of variance was performed to evaluate the experimental error, the significance of each factor, and their interactions on delta-endotoxins production (Table 4). Based on ANOVA, the “*F*-value” for the overall regression model (22.27) is significant at the 1% level. Aeration level significantly increased delta-endotoxins production. Moreover, increasing yeast extract supply allowed to increase bioinsecticides production from 4871.5 to 4966 mg, even with high dissolved oxygen (75% oxygen saturation). Strong catabolite repression was observed at 40 g/L glucose (Table 3). However, at high aeration level (75% oxygen saturation) throughout the fermentation, delta-endotoxin synthesis increased significantly reaching 4871.5 mg. Providing dissolved oxygen saturation of 75% in the medium throughout the fermentation with increased yeast extract concentration of 10.31 g/L led to a high delta-endotoxin production, corresponding to production yield of 76.39 mg/g glucose. In this case, delta-endotoxins production was greatly affected by aeration profile (*P* < 0.01) and yeast extract concentration (*P* < 0.01). However, the statistical analysis did not show any interaction between the three factors (aeration level, glucose, and yeast extract) (Table 4). This finding suggested that the addition of this nitrogen source contributed to improve delta-endotoxin production.

This improvement in bioinsecticides production was not associated to an improvement in cell growth. Production and cell growth illustrated in Table 3 showed that increasing of dissolved oxygen concentration caused an increasing in delta-endotoxin production and that the efficiency in high glucose concentration consumption was hampered under oxygen limiting conditions. Consequently, a high dissolved oxygen concentration increased the cell capacity to metabolize glucose and to produce delta-endotoxins but did not affect the biomass growth rate. Thus, there was no correlation between cell growth and delta-endotoxin production. In fact, an equilibrium should exist between vegetative growth and delta-endotoxin synthesis during last phase of the culture. Delta-endotoxin production improvement could be attributed to the interaction of significant medium compounds which affected the rate of delta-endotoxin synthesis and the size of crystals [28]. Besides, changes in cultural conditions induced different metabolic pathways in* B. thuringiensis *strain that could modify its reserve of energy storing materials [29, 30], which could positively affected toxin production. In this case, the generated energy flux in the catabolism was not utilized for generating new cells (growth) or for maintaining the structural and functional integrity of cells (maintenance), but for delta-endotoxin synthesis.

Such results showed that, into 3 L fermenter, it was possible to overcome, at least partially, the repressive regulation of delta-endotoxin synthesis observed on flask scale using an adequate aeration level. Nevertheless, at 40 g/L glucose, the production yield could be enhanced if dissolved oxygen saturation could be maintained at a level higher than 75%. However, oxygen saturation could not exceed 75% even if the agitation speed and the air flow are applied at their maximal levels in 3 L fermenter. Therefore, more effective aeration systems should be included in the fermenter for the use of higher glucose concentrations. This is related to oxygen transfer capacity of the fermenter. The toxicity against* E. kuehniella* larvae is presented in Table 3. Maximum toxicity was observed at 75% oxygen saturation throughout the fermentation and high glucose concentrations. However, the weakest activity was found in 60–40% oxygen saturation in which delta-endotoxins production had decreased at 30 g/L glucose as well as 40 g/L glucose. These findings indicated the effectiveness of high aeration level in the improvement of the toxicity. These results agreed with those reported by Maldonado-Blanco et al. [31] who obtained the most toxic product at high aeration rates. Moreover, there was a significant negative correlation between delta-endotoxin production and LC_50_ value (correlation coefficient = −0.75***). This means if delta-endotoxin production increases, LC_50_ value will be reduced, which involves an increase in S22* B. thuringiensis* toxicity. Consequently, at high glucose concentrations, the excessive aeration improved not only delta-endotoxin production, but also bioinsecticides toxicity.

### 3.3. Overcome of Catabolite Repression on Delta-Endotoxin Synthesis by Fed-Batch Intermittent Cultures

The major limitation of bioinsecticides production was the repressive regulation of delta-endotoxin synthesis, caused by increased concentrations of readily assimilated carbon sources [9]. It was mostly exhibited during cell growth at the six first hours of incubation [11]. Consequently, the use of FBIC especially during the first ten hours of incubation should ensure growth at low glucose concentrations and then could contribute to the overcome of the repressive regulation. In this context, cultures were performed into 3 L fermenter using glucose and glycerol as carbon and energy sources in the medium (Table 5). The results showed that when steady glucose concentration was maintained at almost 22.5 g/L during the ten first hours of culture, toxin synthesis yield reached 96 mg/g glucose with consumption of 35 g/L glucose. This yield was similar to that obtained by using simple batch (22.5 g/L) with an improvement in toxin production reaching 36%. Interestingly, this yield was similar to that obtained when both glucose and yeast extract concentrations were supplied and maintained constant (Table 5). Moreover, there is not a significant difference in delta-endotoxin production between the two conditions. It could be suggested that nitrogen feeding was not necessary in this strategy of FBIC, since it did not affect toxin production. Moreover, when maintaining glucose concentration at 15 g/L, 97.99 mg toxin/g glucose yield was obtained with 29 g/L glucose. This yield was higher than that obtained with 30 g/L glucose used at simple batch. Besides, the same toxin synthesis yield was reached, when steady glucose concentration was maintained at 10 g/L with total glucose consumption corresponding to 19 g/L (Table 5). These findings confirmed that, at these conditions of FBIC, cells produced during the first ten hours of culture were derepressed concerning delta-endotoxin synthesis compared to those cultured in presence of the total amount of carbon source from the beginning of the culture. Ghribi et al. [11] had reported that it was possible to overcome the catabolite repression, by controlling oxygen in the culture media of* B. thuringiensis*, mostly due to the rate of use of energy generated during a high growth of* B. thuringiensis *cells. In fact, the overcoming of the repressive regulation of delta-endotoxin synthesis, representing the major limitation of bioinsecticides production, was considered as a key step in the overproduction of bioinsecticides by fermentation [9]. This result may be used in practice, since adequate strategy of glucose feeding leading highly concentrated media could conduct a high production of insecticidal crystal proteins, with overcoming of catabolite repression. The final CFU values obtained from the different FBIC fermentations were statistically similar to values obtained in batch culture. This finding is in agreement with the results of Avignone Rossa and Mignone [32], who, using fed-batch culture, obtained similar biomass production by batch and fed-batch cultures. However, this result is different from that of Kang et al. [33] who, using FBIC, increased biomass production. Consequently, the applied fed-batch strategy greatly improved delta-endotoxin production and the cell's capacity of delta-endotoxins synthesis (Table 5) without increasing cell growth.

Several FBIC showed promising results when used to improve biomass and consequently spore production of* B. thuringiensis* [33, 34]. However, no works had been done on the optimization of delta-endotoxin production by fed-batch strategy. The importance of such fed-batch technology was especially illustrated when using high glucose concentrations. Indeed, with 42.5 g/L glucose provided progressively during the first fifteen hours of the culture, toxin production reached 90 mg toxin/g glucose which was clearly more important than that obtained even with 40 g/L initial glucose concentration in the culture medium (Table 5). In addition, toxin synthesis by cells was clearly improved when using fed-batch culture rather than simple one. In fact, toxin synthesis yield was clearly increased from 62.23 to 96.75 mg toxin/10^10^ cells, when using 40 g/L and 42.5 g/L carbon source, respectively. This result could be explained by the fact that cell growth was not improved when using fed-batch conditions. Indeed, using, respectively, simple batch culture and FBIC, final CFU titers were statistically similar (Table 5). These results confirmed that such cells were derepressed and could overproduce delta-endotoxins during stationary phase. Consequently, toxin production yield per cell was markedly increased (Table 5).

Bioassay results indicated that FBIC affected* B. thuringiensis *toxicity. Indeed, toxicity data (Table 5) showed a significant decrease in LC_50_ values according to Duncan test, when applying the fed-batch strategy, compared to those obtained by simple batch. However, Roy et al. [35] and Avignone Rossa and Mignone [32] reported that the insecticidal activity obtained with fed-batch or continuous culture was lower than the batch culture. Consequently, in this case, FBIC allowed to improve not only delta-endotoxin production and yield but also insecticidal activity of* B. thuringiensis *bioinsecticides.

## 4. Conclusion

The overcoming of catabolite repression which regulated delta-endotoxin production was considered as a key step in the overproduction of insecticidal crystal protein by fermentation. Firstly, by an efficient supply of oxygen which was considered one of the most important factors in the scale-up of fermentation processes, it was possible to partially overcome the catabolite repression of delta-endotoxin synthesis. Secondly, the original fed-batch strategy proposed could well overcome the catabolite repression during the fermentation process. Therefore, the improved fed-batch strategy, which considered the suitable glucose supply by earlier feeding, greatly improved the toxin production yield and the cell's capacity of delta-endotoxin synthesis. Thus, it was possible to overproduce delta-endotoxins using high carbon source amounts, through applying adequate fermentation technology. The determination of biological activity could be one of the most important factors for optimizing bioinsecticides production. This should contribute to the reduction of sporeless* B. thuringiensis* bioinsecticides production costs.

## Figures and Tables

**Table 1:** Independent variables and their levels for the 2^3^ factorial experimental designs.

Factor	Name	Unit	Coded level	
			−1	+1
*X*_1_	Aeration profile	%	60–40	75-75
*X*_2_	Glucose	g/L	30	40
*X*_3_	Yeast extract	g/L	5.8	10.31

**Table 2:** Aeration effect on delta-endotoxin production in glucose based medium.

Aeration profiles (% oxygen saturation)	Total production (mg)	CFU (10^7^ cells/mL)	Toxin synthesis yield (mg/10^10^ cells)∗∗	Toxin production yield (mg/g glucose)∗∗∗	LC_50_ (ng of toxin per mg of flour)
60–40	4338 ± 130.7^a*^	18 ± 4.3^a^	120.5	113.41	560.86 ± 8.59^bc^
70–40	3704 ± 90.5^b^	15 ± 3^a^	120.46	96.83	574.65 ± 13.06^b^
60–30	4202 ± 146.6^a^	20 ± 3.6^a^	105.05	109.85	546.61 ± 15.16^c^
40-40	3064 ± 26.5^d^	17 ± 2.6^a^	97.41	80.1	604.09 ± 7.09^a^
60–50	3430 ± 98.2^c^	15 ± 2.6^a^	114.33	89.67	592.54 ± 3.29^a^

*R* ^2^ = 0.97; coefficient of variance = 2.84; [(Pr > *F*) < 0.001].

∗The letters a, b, c, and d used as indices in the same column indicated the presence of significant differences (Duncan's multiple range test, *P* < 0.05) among the experiments, in ascending order.

∗∗Relative toxin yield calculated as the ratio of delta-endotoxins (mg/L) over CFU (10^10^ cells/L) both of them determined at the end of fermentation.

∗∗∗Toxin production yield calculated as the ratio of delta-endotoxin (mg/L) over assimilated glucose (g/L).

**Table 3:** Aeration effect on delta-endotoxins production in media based on different concentrations of glucose and yeast extract.

Aeration profiles	Glucose (mg/L)	Yeast extract (g/L)	Total production (mg)	CFU (10^7^ cells/mL)	Toxin synthesis yield (mg/10^10^ cells)	Toxin production yield (mg/g glucose)	LC_50_ (ng of toxin per mg of flour)
60–40	30	5.8	4005 ± 117.37^d*^	22 ± 2.82^f^	107.08	78.52	604.8 ± 2.99^a^
75-75	30	5.8	4871 ± 148.49^b^	39 ± 5.65^c,d^	73.46	95.5	574 ± 5.65^b,c^
60–40	40	5.8	4107 ± 111.72^c,d^	26 ± 5.65^e,f^	92.91	60.39	592.11 ± 24.52^a,b^
75-75	40	5.8	4871.5 ± 149.19^b^	44 ± 0^b,c,d^	65.12	71.63	557.88 ± 5.35^c^
60–40	30	10.31	4382.5 ± 38.89^c^	35 ± 1.41^d,e^	73.65	85.93	579.44 ± 4.76^a,b,c^
75-75	30	10.31	4966 ± 106^a,b^	48 ± 2.82^b,c^	60.85	97.37	554.72 ± 0.88^c^
60–40	40	10.31	4356 ± 31.11^c^	51 ± 7.07^b^	50.24	64.05	580 ± 11.31^a,b,c^
75-75	40	10.31	5195 ± 145.66^a^	66 ± 5.65^a^	46.3	76.39	556.39 ± 7.46^c^

*The letters a, b, c, d, e, and f used as indices in the same column indicated the presence of significant differences (Duncan's multiple range test, *P* < 0.05) among the experiments, in ascending order.

**Table 4:** ANOVA results for the three factorial designs for delta-endotoxins production.

Source	Sum of squares	DF	Mean square	*F*-value	Pr > *F*
Model	2677861	8	334732.625	22.27	<0.01∗∗∗
*X*_1_	2330202.25	1	2330202.25	155.01	<0.01∗∗∗
*X*_2_	23256.25	1	23256.25	1.55	0.2536
*X*_3_	273006.25	1	273006.25	18.16	<0.01∗∗∗
*X*_1_ × *X*_2_	5929	1	5929	0.39	0.5499
*X*_1_ × *X*_3_	10816	1	10816	0.72	0.4244
*X*_2_ × *X*_3_	2500	1	2500	0.17	0.6956
*X*_1_ × *X*_2_ × *X*_3_	31862.25	1	31862.25	2.12	0.1888
Pure error	105230	7	15032.857		

*R* ^2^ = 0.962; Adj *R* ^2^ = 0.926; CV = 2.66					

***indicates significance at the level 99.9%.

**Table 5:** Effect of glucose feeding on production of bioinsecticides into 3 litre fermenter.

Initial concentration of glucose (g/L)	Total glucose consumption (g/L)	Yeast extract (g/L)	Total production (mg)	CFU (10^7^ cells/mL)	Toxin synthesis yield (mg/10^10^ cells)	Toxin production yield (mg/g glucose)	LC_50_ (ng of toxin per mg of flour)
22.5∗∗	22.5	5.8	4202 ± 103.5^d*^	22 ± 4.5^a^	94.54	109.85	626.87 ± 24.03^a^
22.5	35	5.8	5712 ± 141.7^b^	25 ± 7.9^a^	95.2	96	553.35 ± 11.91^c^
22.5∗∗∗	34.07	10	5650 ± 70.3^b^	26 ± 8.1^a^	94.46	97.55	542.32 ± 9.26^c^
10	18.77	5.8	3126 ± 16^e^	15 ± 5.2^a^	94.66	97.96	561.41 ± 5.69^c^
15	29	5.8	4831 ± 150^c^	22 ± 9.1^a^	99.81	97.99	556.29 ± 14.37^c^
30	30	5.8	4035 ± 106.8^d^	22 ± 11.5^a^	83	79.11	620.37 ± 18.45^a^
40	40	5.8	4107 ± 81^d^	30 ± 6^a^	62.23	60.39	589.85 ± 17.81^b^
22.5	42.5	5.8	6503 ± 49.1^a^	28 ± 7.5^a^	96.75	90	552.61 ± 11.55^c^

*R* ^2^ = 0.994; coefficient of variance = 2.17; [(Pr > *F*) < 0.001].

∗The letters a, b, c, d, and e used as indices in the same column indicated the presence of significant differences (Duncan's multiple range test, *P* < 0.05) among the experiments, in ascending order.

∗∗Batch culture curried out with the mentioned carbon source and yeast extract concentrations.

∗∗∗Fed-batch culture realized with feeding both glucose and yeast extract.
